# An intermediate significant bit (ISB) watermarking technique using neural networks

**DOI:** 10.1186/s40064-016-2371-6

**Published:** 2016-06-24

**Authors:** Akram Zeki, Adamu Abubakar, Haruna Chiroma

**Affiliations:** Department of Information Systems, International Islamic University Malaysia, P.O. Box 10, 53100 Jalan Gombak, Kuala Lumpur, Malaysia; Department of Artificial Intelligence, University of Malaya, 50603 Pantai Valley, Kuala Lumpur, Malaysia

**Keywords:** Peak signal-to-noise ratio, Bit error rate, Normalised cross-correlation, Intermediate significant bit

## Abstract

Prior research studies have shown that the peak signal to noise ratio (PSNR) is the most frequent watermarked image quality metric that is used for determining the levels of strength and weakness of watermarking algorithms. Conversely, normalised cross correlation (NCC) is the most common metric used after attacks were applied to a watermarked image to verify the strength of the algorithm used. Many researchers have used these approaches to evaluate their algorithms. These strategies have been used for a long time, however, which unfortunately limits the value of PSNR and NCC in reflecting the strength and weakness of the watermarking algorithms. This paper considers this issue to determine the threshold values of these two parameters in reflecting the amount of strength and weakness of the watermarking algorithms. We used our novel watermarking technique for embedding four watermarks in intermediate significant bits (ISB) of six image files one-by-one through replacing the image pixels with new pixels and, at the same time, keeping the new pixels very close to the original pixels. This approach gains an improved robustness based on the PSNR and NCC values that were gathered. A neural network model was built that uses the image quality metrics (PSNR and NCC) values obtained from the watermarking of six grey-scale images that use ISB as the desired output and that are trained for each watermarked image’s PSNR and NCC. The neural network predicts the watermarked image’s PSNR together with NCC after the attacks when a portion of the output of the same or different types of image quality metrics (PSNR and NCC) are obtained. The results indicate that the NCC metric fluctuates before the PSNR values deteriorate.

## Background

The quality of an image presentation is improved by image enhancement techniques, which are used to present the best outlook of a captured scene (Zeki and Manaf 2009; Hua et al. 2016). However, this approach can be judged by the human visual system; in such cases, the results of a subjective study would be helpful when the majority of the participants indicate that there is a better quality presentation (Rahman et al. 2011). This strategy will help to determine the quality of the image within some ranges provided by the participants’ feelings about it. Next, the method that was used in the image enhancement will now be known to be the best method for producing a high-quality image presentation. Image enhancement or processing techniques are performed either in the spatial domain or in the transform domain (Yalman and Erturk 2013). The former involves the direct manipulation of the image pixels, whereas in the transform domain techniques, the image is transformed into its frequency domain first usually by a Fourier Transform or by other transformation methods. Then, all of the manipulations will be conducted on the transform image. The final result will be the Inverse transform of the image. The result of image enhancement always provides a better presentation. Consider a general image enhancement technique, where $x$ represents an original image (let us assume a grey-scale image). The new image that will be produced after enhancement will not be the same as the original image $x$; assume that $y$ is the new image that will be formed after the enhancement. Then, $x$ will first be transformed into $y$ by a transformation function $z$ if $p$ and $q$ represent the pixel values of $x$ and $y$ respectively, which means that the pixel values of $q$ result from the transformation of the pixel values of $x$ by altering the original image with the transformation function, which can be represented as $q = z(p)$. This approach involves the utilisation of all the pixels contained in $x$. Because *x* is a grey-scale image, the range will be $\left[ {0,255} \right]$ depending on the filled and free space of this range, and the number of bits $n$ that will be used is in the range $\left[ {0,n - 1} \right]$.

To further validate the quality of the image enhancement method without relying on only a subjective technique, quantitative values are required to show the extent to which certain methods of image enhancement perform the best (Bhatnagar et al. 2012). This knowledge will solve the problem of determining that one method of image enhancement provides a better quality image than another. However, a quantitative approach used in determining the quality of an image focuses on the level of noise (an undesired part introduced randomly on the useful part of the image) in the image, which is the ratio of the noise and the useful information. This approach will measure and compare the effects of the image enhancement techniques on the image quality. Technically, this approach is described as PSNR (Ji et al. 2013; Hsu and Hu 2015; Ouyang et al. 2015).

Unfortunately, while attempting to perform image processing/enhancement as the key for quality measurement, in the field of image watermarking the effect of image enhancement/processing techniques results in what is known as an attack (Akar et al. 2013). The reason is that in image watermarking, an additional bit is embedded in either the transformation or spatial domain of the image, to prevent the original image from being reproduced by unauthorised persons (Duda et al. 2001). Thus, applying an image enhancement or processing operation on a watermarked image could alter or damage the watermarked bits. Any watermarking technique that can withstand these image enhancement or processing techniques and maintain the quality of the image after embedding a watermark on it is described as a robust watermarking technique (Briffa and Das 2003; Syed et al. 2006; Gilani and Skodras 2002). Over the years, researchers have been designing watermarking techniques with robustness in mind, in order for the watermark to be resistant against any image processing technique, although a necessary condition for the robust watermarking technique still remains with the quality of the technique itself (Matt and Jeffrey 1999). Furthermore, the requirement of a good watermarking technique includes a tradeoff between robustness, image quality (imperceptibility) and capacity. Moreover, it has been found that when the robustness of the watermarking method improves, the imperceptibility decreases, and the capacity increases (Rahman et al. 2011; Yalman and Erturk 2013; Ji et al. 2013; Akar et al. 2013; Duda et al. 2001).

Different parameters are used to assess the robustness of watermarking, namely bit correct ratio (BCR) (Maity and Kundu 2002; Hua et al. 2016), or the bit error ratio (BER) (Parameswaran and Anbumani 2006; Zhang et al. 1998). In some cases, similarity was used to draw some conclusion (Bishop 2006), whereas the probability approach is also considered as a robustness measure in Lu (2005). Robustness measures through these methods give almost the same result. Consistent with this, normalised cross correlation (NCC) has been used in the current study. This famous technique is mostly used as a parameter for testing the robustness of the watermarking (Kiani and Ebrahimi 2011; Braudaway et al. 1996). The issue raised by this researcher is the extent to which these quality measures relate to determining the quality of a watermarked file after embedding and after it has undergone an attack. This paper presents an investigation with a neural network (NN) model on the watermarked image quality metric (PSNR and NCC) values obtained from the watermarking of six grey-scale images using ISB. The watermarking technique used involves the embedding of a watermark in intermediate significant bits (ISB) (Hal 1997). There are several types of NN, including the recurrent, generalised regression. Multi-layer perceptron NN (MLPNN) is the most widely used NN for prediction (Yeung and Mintzer 1997), and it performs better than other NNs in terms of its accuracy in recognising patterns (Bender et al. 1996); therefore this study has chosen to use it. The neural network model used the image quality metric (PSNR and NCC) values obtained from the watermarking of six grey-scale images that used ISB as the desired output and trained the network to predict future values when some output of the same or different type of image quality metrics (PSNR and NCC) were obtained.

### Robustness of the watermarking techniques

Many image watermarking algorithms have been presented by various researchers with the aim of preventing the ownership of the image and for copyright protection. Digital watermarking in general is a special case of the general information-hiding problem (Wójtowicz and Ogiela 2016). A digital watermark is a signal that is temporarily or permanently embedded into digital data (audio, images, videos and text), which can be detected or extracted later by means of a computing operation, to make an assertion about the ownership of the data. The watermark is hidden in the host data, in such a way that it is inseparable from the data and resistant to image processing operations, and it does not degrade the host file. Thus, by means of watermarking, the real image is still accessible but is permanently marked (Caronni 1995; Liu et al. 2016). Image watermarking can be classified as visible or invisible. A visible watermark typically contains a visual message or a company’s logo, which indicates the ownership of a specific image. An invisible watermarked image is visually very similar but not necessarily identical to the original unmarked image. A substantial amount of work has been conducted on visible image watermarking (Jin and Chang 2004; Lin et al. 2010), as well as on invisible watermarking (Bas et al. 2002). The existence of an invisible watermark can be determined only through a watermark extraction or detection algorithm. Invisible watermarking, however, is a far more complex concept. It is most often used to identify copyrighted data, such as by the author, distributor or others.

The robustness of watermarking techniques has received a large amount of attention among researchers because it reflects good performance of certain techniques and shows how resistant to attack a technique can be. A robust watermarking technique prevents a watermark attack against geometric distortions, ensures the synchronisation of the watermark before and after embedding, and ensures watermark resilience to common image processing attacks as well as desynchronisation attacks (Huo-Chong et al. 2011; Mardanpour and Chahooki 2016). Robust watermarking ensures self-synchronising schemes, which will certainly permit the recovery of the watermark after geometrical attacks (Li et al. 2006). A robust watermarking technique can ensure the mapping of original watermark image features with the watermarked image features to be resilient against the image processing, such as affine transformations (Hong-ying et al. 2013; Liu et al. 2006). Although there are other watermarking techniques that are not robust, which are affected by many attacks, the intention might clearly be that any attempt to make an alteration should damage the image. In that case, the technique serves its purpose. Any watermarking technique with robustness in mind should tend to be resistant against image processing operations, utilising any concept that is necessary. For example, a mathematical remainder operation might be used to build a robust watermarking technique that will modify the low-frequency coefficients in the DCT frequency domain (Rafi et al. 2013). However, it has been observed that, in some cases, an image file that has not undergone any watermarking process shows to an attacker that the image file contains a watermark (Javier and Angel 2007); this scenario could mean that the attacking techniques are sensitive. In other approaches, watermarking techniques over a network should be based on partial encryption, which will decrease computation and bandwidth consumption in an effort to increase the robustness (Fan et al. 2013). However, some techniques are used to overcome sophisticated attacking techniques, to improve the robustness by using a fuzzy support vector machine (FSVM) correction to learn the geometric distortion parameters and apply low-order Zernike moments (Voloshynovskiy et al. 2001). Here, a self-reference image will be used and then transformed into the original image in the wavelet domain, and the watermark is embedded by taking advantage of the different values of the two images (Hanjalic et al. 2000). The robustness of watermarking techniques is essential in hardware-based watermarking techniques. The correlation of the original watermark image and the watermarked image makes it capable of detecting the inserted bits (Hartung and Kutter 1999; Jiao et al. 2015). For this reason, it can be implemented in a hardware-oriented image coding processing scheme (Kougianos et al. 2009), while using any transformation technique. At the same time, the relationship between the capacity and the bit error rate will maintain at a lower level (Juergen 2005; Abbasi et al. 2015).

### Intermediate significant bit (ISB) watermarking technique

**Fig. 1:**
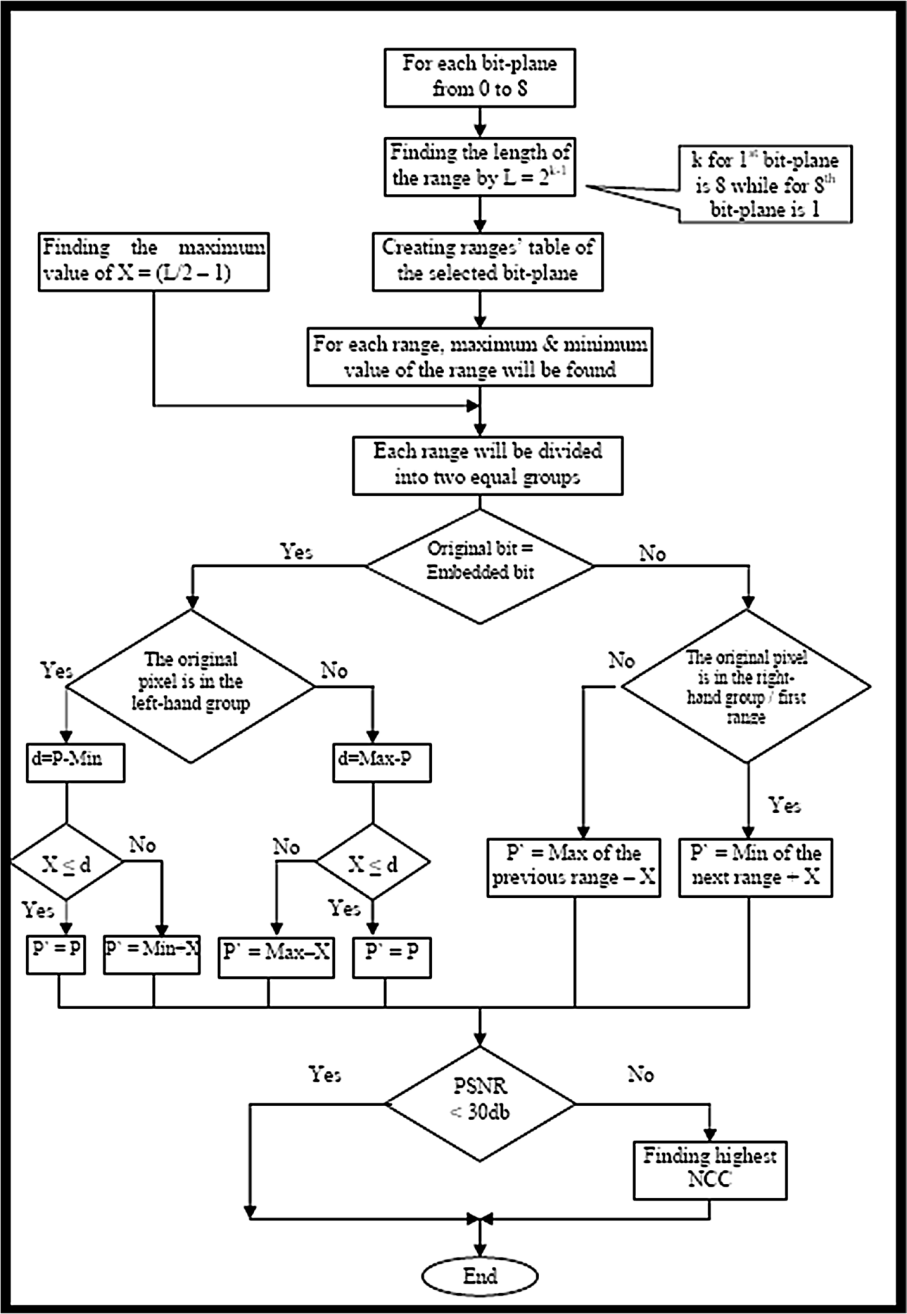
Step-by-step procedure of the ISB technique

An intermediate significant bit (ISB) embedding technique was proposed with robustness in mind (Onur and Elbasi 2014; Yahya et al. 2015). The technique replaces the original image pixels within an empty/filled region with watermark pixels “at the same time, keeping the watermark pixels very close to the filled region of the original image pixels” (Onur and Elbasi 2014). “The technique was based on testing the value of the watermark pixel according to the range of each bit-plane and positioning the original image file pixel away from any of the edges of the range”. A bit-plane of digital images is a set of bits that have the same position in the respective pixels of the digital image. In the representation of grey-scale images, there are eight bit-planes: the first bit-plane contains the set of the most significant bits, while the 8th contains the least significant bits, and the set in between, from the 2nd to the 7th bit-planes, are intermediate significant bits (ISB). The best pixel value between the middle and the edge of the range (threshold value) has been found to protect the watermarked object from the different types of attack and keep the minimum distortion of the watermarked image. Embedding the information based on the blocks of a few pixels will improve the robustness of the model, especially against the geometrical transform attacks. The security of the system will be improved by encrypting the watermark object using the random pixel manipulation technique, before being embedded within other host images. In addition, the capacity will be improved using the pixel value difference method to embed more data into edge areas, as the human visual system is less sensitive to distortions around the edge areas than in the smooth areas. The technique follows the stages presented in the flow chart in Fig. 1.

Let us assume that the original pixel value *P* is defined by Eq. 1, below:

1 $$P = \sum\limits_{i = 1}^{8} {y_{i} 2^{i - 1} }$$

where $y_{i}$ is the value of each bit-plane in the binary form (0 or 1), and $i$ ranges between 1 and 8; $i$ = 1 for the LSB and $i$ = 8 for MSB. Assume that the selected bit for embedding is $k$, and the embedded bit is $b$, which is 0 or 1. The new watermarked pixels $P^{\prime}$ can be defined as follows: if the embedded bit is equal to the original bit $\left( {y_{k} = b} \right)$, the watermarked pixel is then equal to the original pixel, as given in Eq. 2 below:

2 $$P^{\prime} = P\quad if\;y_{k} = b$$

In a situation in which the embedded bit is not equal to the original bit $\left( {y_{k} \ne b} \right)$, and the original pixel is located in the first range $\left( {P < 2^{k - 1} } \right)$, then the watermarked pixels can be obtained from Eq. 3.

3 $$P^{\prime} = 2^{k - 1} \quad if\;y_{k} \ne b\quad and \quad P < 2^{k - 1}$$

If the original pixel is located in the last range $\left( {P > 2^{8} - 2^{k - 1} } \right)$, then the watermarked pixels can be obtained by using Eq. 4.

4 $$P^{\prime} = \sum\limits_{i = 1}^{8} {y_{i} 2^{i - 1} - 1} \quad if\;y_{k} \ne b\quad and \quad P > 2^{8} - 2^{k - 1}$$

When the original pixel is neither in the first range nor in the last range, the watermarked pixels can be obtained from Eq. 5.

5 $$P^{\prime} = \left\{ {\begin{array}{*{20}l} {\sum\nolimits_{i = k + 1}^{8} {y_{i} 2^{{i - 1^{k} }} } + b \times 2^{k - 1} - 1\quad if\;y_{k} \ne b\quad and\quad y_{k - 1} = 0} \hfill \\ {\sum\nolimits_{i = k + 1}^{8} {y_{i} 2^{i - 1} } + 2^{k - 1} + b \times 2^{k - 1} \quad if\;y_{k} \ne b\quad and \quad y_{k - 1} = 1} \hfill \\ \end{array} } \right.$$

To improve the quality of the watermarked image of the bit-plane model, the watermark bits were inserted into the selected bit, and the other seven bits were changed to closely assimilate the original pixel. This action was performed by moving the pixel to the location at the edge of the ranges toward the original pixel. The quality of the watermarked images, using the proposed method, was found to an improvement over the LSB method, except for the quality of the 8th bit-plane (the least significant bits). The merit of the proposed method over the LSB method is that it was found to gradually increase by moving it from the 8th bit-plane to the 1st bit-plane. Assume that *P* is the original pixel value, *P′* is the new watermarked pixel, *k* is the selected bit for embedding, *u* is the value of all bit-planes to the right of the selected bit-plane k, *i* is the bits [starting from the bit next to the concerned bit (*k*) until the last bit *i*, which ranges from 1 to 8, *i* = 1 for LSB and *i* = 8 for MSB], *b* is the embedded bit (which is 0 or 1), and finally, *X* is the shift value (which is known as the bias value) to protect against attacks. Using Eq. 1, we obtain

6 $$u = \sum\limits_{i = 1}^{k - 1} {y_{i} 2^{i - 1} }$$

In this case, the algorithm will respond to any changes within the ranges; for example if the embedded bit is equal to the original bit $y_{k} = b$ and the original pixel is less than $2^{k - 1}$, which is located in the first range, the watermarked pixel can be obtained from Eq. 7, as follows:

7 $$P^{\prime} = \left\{ {\begin{array}{*{20}l} {2^{k - 1} - X - 1\quad if \quad u > 2^{k - 1} - X - 1} \hfill \\ {u\quad if \quad u \le 2^{k - 1} - X - 1} \hfill \\ \end{array} } \right.$$

If the embedded bit is not equal to the original bit $y_{k} \ne b$, then the watermarked pixel can be obtained by using Eq. 8:

8 $$P^{\prime} = 2^{k - 1} + X$$

In the situation in which the original pixel is greater than $2^{8} - 2^{k - 1}$, which is located in the last range, and if the embedded bit is equal to the original bit $y_{k} = b$, then the watermarked pixel can be obtained by using Eq. 9:

9 $$p^{\prime} = \left\{ \begin{aligned} u + \sum\nolimits_{i = k}^{8} {y_{i} 2^{i - 1} \quad if\quad u > X} \hfill \\ X + \sum\nolimits_{i = k}^{8} {y_{i} 2^{i - 1} \quad if \quad u \le X} \hfill \\ \end{aligned} \right.$$

If the embedded bit is not equal to the original bit $y_{k} \ne b$, then the watermarked pixel can be obtained by using Eq. 10:

10 $$P^{\prime} = \sum\limits_{i = k}^{8} {y_{i} 2^{i - 1} - X - 1}$$

In a situation in which the value of the original pixel is not very high or very low (in other words, the pixel value is in neither the lowest range nor the highest range), and if the embedded bit is equal to the original bit $y_{k} = b$, then the watermarked pixel can be obtained as given in Eq. 11:

11 $$P^{\prime} = \left\{ {\begin{array}{*{20}l} {\sum\nolimits_{i = k}^{8} {y_{i} 2^{i - 1} } + 2^{k - 1} - X - 1 \quad if \quad u > 2^{k - 1} - X - 1} \hfill \\ {\sum\nolimits_{i = k}^{8} {y_{i} 2^{i - 1} } + X\quad if\;u < X} \hfill \\ {\sum\nolimits_{i = k}^{8} {y_{i} 2^{i - 1} } + u\quad if\;u \ge X\quad and \quad u \le 2^{k - 1} - X - 1} \hfill \\ \end{array} } \right.$$

If the embedded bit is not equal to the original bit $y_{k} \ne b$, then the original bit is equal to one and the embedded bit is zero ($y_{k} = 1\;\;and\;\;b = 0$); then, the watermarked pixel can be obtained from Eq. 12:

12 $$P^{\prime} = \left\{ {\begin{array}{*{20}l} {\sum\nolimits_{i = k + 1}^{8} {y_{i} 2^{i - 1} } + 2^{k - 1} - X - 1\quad if\quad y_{k - 1} = 0} \hfill \\ {\sum\nolimits_{i = k + 1}^{8} {y_{i} 2^{i - 1} } + 2^{k} + X\quad if\quad y_{k - 1} = 1} \hfill \\ \end{array} } \right.$$

If the embedded bit is not equal to the original bit $y_{k} \ne b$, then the original bit is equal to zero and the embedded bit is one ($y_{k} = 1\;\;and\;\;b = 1$); the watermarked pixel can be obtained as in Eq. 13:

13 $$P^{\prime} = \left\{ {\begin{array}{*{20}l} {\sum\nolimits_{i = k + 1}^{8} {y_{i} 2^{i - 1} } - X - 1\quad if\quad y_{k - 1} = 0} \hfill \\ {\sum\nolimits_{i = k + 1}^{8} {y_{i} 2^{i - 1} } + 2^{k - 1} + X\quad if \quad y_{k - 1} = 1} \hfill \\ \end{array} } \right.$$

Equations 12 and 13 can be rewritten to form Eq. 14, by inserting *b* as the embedded value (which is either 0 or 1) into the *k*-bit plane:

14 $$P^{\prime} = \left\{ {\begin{array}{*{20}l} {\sum\nolimits_{i = k + 1}^{8} {y_{i} 2^{i - 1} } + b2^{k - 1} - X - 1\quad if\quad y_{k - 1} = 0} \hfill \\ {\sum\nolimits_{i = k + 1}^{8} {y_{i} 2^{i - 1} } + 2^{k - 1} + b2^{k - 1} + X\quad if\quad y_{k - 1} = 1} \hfill \\ \end{array} } \right.$$

**Fig. 2:**
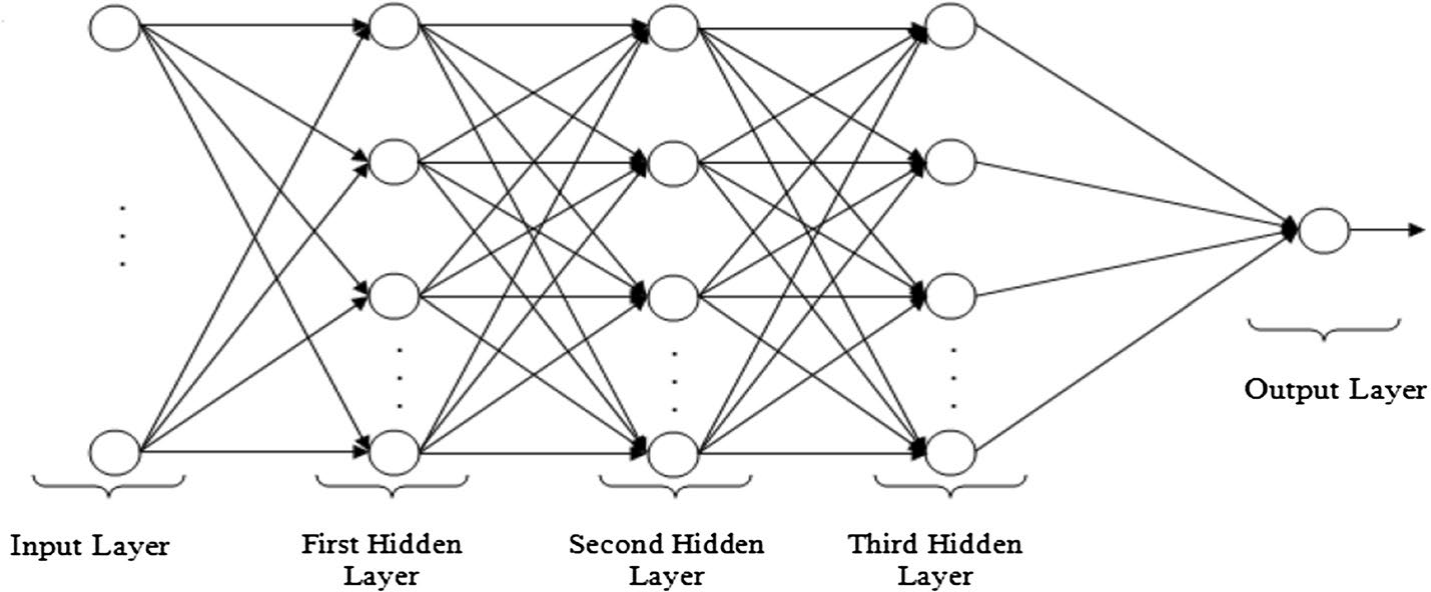
The Block diagram of a three hidden layer multilayer perceptron (MLP)

The neural network (NN) model is introduced to yield a collective phenomenon for watermarked image quality metrics in which the image undergoes seven so-called watermarking attacks (image processing operations). The fact remains that image quality assessment is important, especially in regard to finding the perfect image. It is well known that the application of a neural network comes from the desire to develop an artificial system that could perform ‘intelligent’ tasks similar to those performed by the human brain. In other words, a network model could gain knowledge through learning and store it in such a way that the knowledge could be applied when the need arises. Conventional linear models obviously are not suitable for modelling data with non-linear attributes. Neural network models are capable of treating both linear and non-linear relationships by learning those relationships directly from the data that is being modelled. Many neural network models are available, among them the multi-layer perceptron NN (MLPNN) model, which is very good at prediction (Shamseldin et al. 2007) and has a high degree of accuracy in recognising patterns (Darus and Al-Khafaji 2012), Previous research has shown that some other NN model are used in watermarking (Wang et al. 2015; Agarwal et al. 2013; Maity et al. 2014; Singh et al. 2016). A graphical representation of a typical MLPNN with three hidden layers is shown Fig. 2, which presents a three hidden layers. The inputs are fed into the input layer and are multiplied by interconnection weights as they are passed from the input layer neurons to the first hidden layer. Within the first hidden layer, the signals are summed, and a nonlinear activation function is computed. As the processed data leaves the first hidden layer, it again gets multiplied by interconnection weights and is then summed and computed by the second hidden layer. As the processed data leaves the second hidden layer, again it gets multiplied by interconnection weights and is then summed and processed by the third hidden layer. Finally, the data are multiplied by interconnection weights and then is processed one last time within the output layer to produce the MLPNN output (Agilandeeswari and Ganesan 2015).

### ISB embedding and extracting of the watermarks

Employing the effects of the bit-plane technique, four watermarked grey-scale images of size 90 × 90 pixels and six host images containing 256 × 256 pixels each (see Fig. 2) were used for the intermediate significant bit embedding technique.

Before the embedding process, the first step is to encrypt the watermark images using the Random Pixel Manipulation Technique, a chosen key string that is effectively manipulated to obtain a random number sequence. The key provides a seed value, which is an integer that helps to generate a repeated sequence of unique pseudo-random numbers that range from 0 to N, where N is the number of available pixels. This sequence is then used to ‘scramble’ the hidden data. At the receiving end, the key is used to uncover the data (it plays the role of a password). It provides the same seed value and consequently the same sequence of unique random numbers as generated at the sender’s side. Therefore, the four watermark images were embedded into the six host images one-by-one using the ISB embedding technique, and the embedded data were distributed randomly throughout the image and could be recovered bit-by-bit, packed and re-grouped to fully regenerate the hidden original data.

The watermark images were embedded into the host images by using the proposed ISB algorithm (at X = 0) for all of the bit-planes, starting from the 1st bit-plane (the most significant bits—MSB) through the 8th bit-plane (the least significant bits—LSB). The peak signal-to-noise ratio PSNR was then calculated. Visual inspection of the watermarked image compared to the PSNR value, for both the LSB and ISB techniques, increased gradually from the 1st bit-plane to the 8th bit-plane. On the other hand, the quality of the watermarked images for the ISB techniques was improved.

The watermarking experiment involves embedding and extracting watermarks on grey-scale images. The experiment is aimed at testing the strengths of embedding in intermediate significant bits (ISB) to evaluate the distortion metrics on the watermark in the watermarked file and the degradation of the watermarked rates. This approach could lead to establishing limiting values for the relationships between them. To demonstrate the effect of each level in the grey-scale images, the host image eight bit-planes are used, starting from the 1st bit-plane in the top left image to the 8th bit-plane in the bottom right image. Observing these bit-planes after removing k bit-planes, it can be observed that some areas in the 6th, 7th and 8th LSB planes are covered with noise. Theoretically, the worst case in the simple LSB substitution method occurs when the watermark bit and original bit are different all the time. In other words, the difference between the original pixel and the watermarked pixel is (2k − 1), where k is the level of the different bit-planes.

**Fig. 3:**
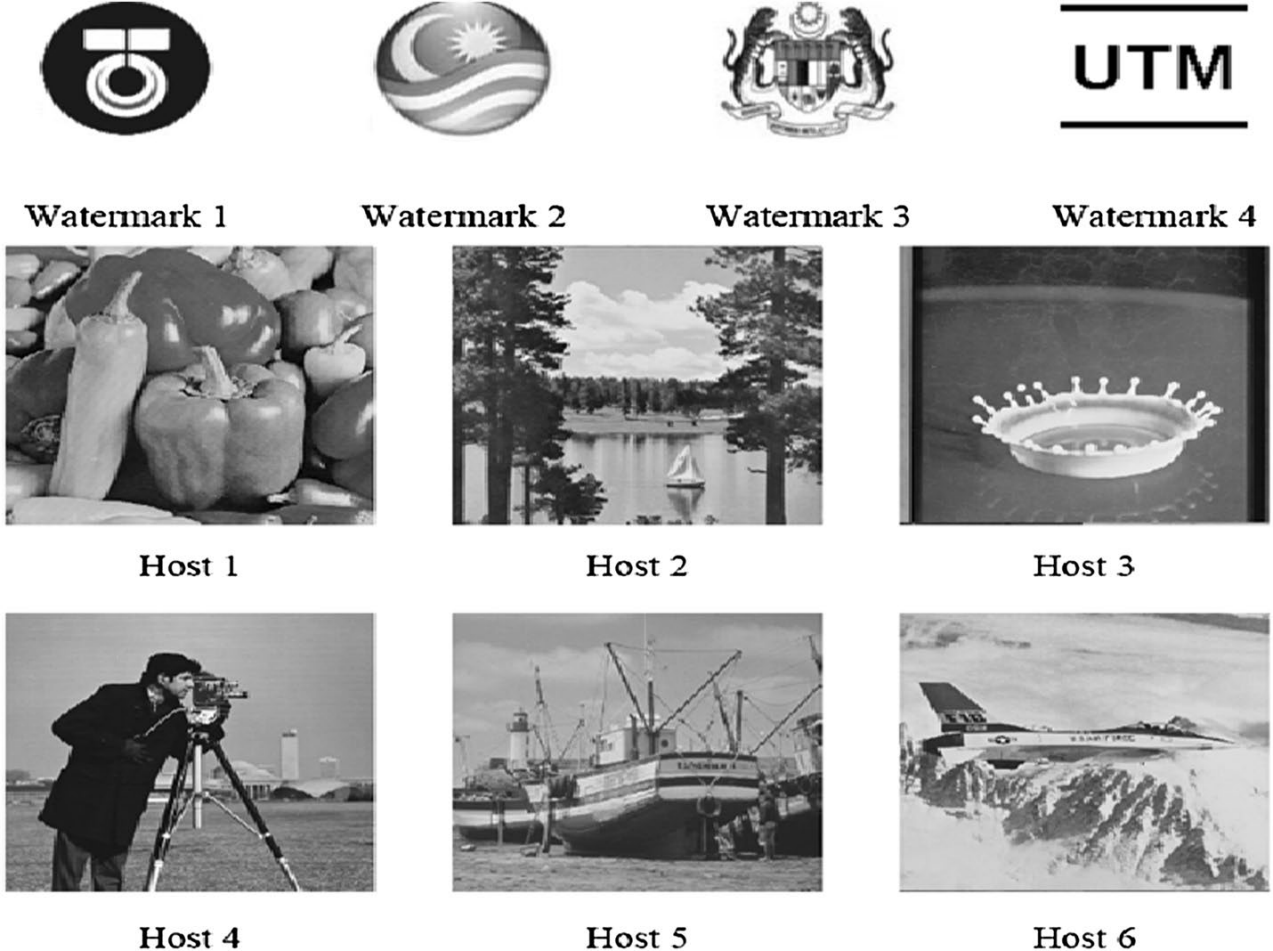
The grey-scale watermark images (90 × 90 pixels each) and host images (256 × 256 pixels each)

PSNR for the worst case of embedding one bit within k bit-planes and embedding the watermark within k-rightmost of the host image are presented in Fig. 3, in addition to the most common distribution embedding for the k bit-plane. Notice that the worst case of the k-rightmost bit-plane can be obtained only when they are all zeros and the embedded bits are ones or the original bits are ones and the embedded bits are zeros.

The above figure shows three PSNR values for different k bit-planes. The first is the worst case, from embedding in the k bit-plane only. The second is for embedding the watermark into the k bit-plane only for the most common case, and the third case is for embedding the worst case into the k-rightmost bit-planes. It can be observed that the image quality of the watermarked image had been drastically degraded when k > 4, in the case of using one bit-plane only for hosting the watermark. In the case of k right-most bit-planes, the image quality of the watermarked image was drastically degraded when k > 3.

**Fig. 4:**
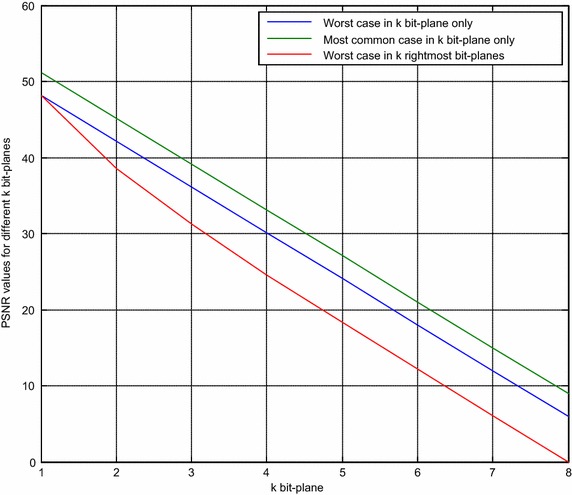
PSNR values for different k bit-planes

The demonstration of the proposed MLPNN model application is shown in Fig. 4. The watermarked file is subjected to seven different attacks and then it was converted from an image format into a binary format (0 and 1). The binary data are then fed into the MLPNN model, which has been trained to make the association between the character image data and a numeric value that corresponds to the character of the image processing and enhancement metric (PSNR and NCC). The output is to determine the limiting values that coexist between the image quality metrics. The network detects patterns in the experimental data in such a way that it can make predictions with reasonable accuracy on the test datasets, similar to the study performed in Agarwal et al. (2015).

### The image quality metrics (PSNR and NCC)

In many successful watermarking algorithms, there is always an absence of any perpetual degradation, and a Peak Signal-to-Noise Ratio (PSNR) is always obtained in excess of 40 dB. This arrangement indicates that little or no noise is being detected after embedding a watermark in the original image file. Furthermore, following an application of some attacks (image enhancement/processing operation), a normalised cross-correlation examination indicates no significant difference between the original image before embedding and after embedding. In contrast to this finding, it has been stated that the PSNR measurement is not valid or it does not truly indicate the signal to noise ratio accurately when the content of the original file changes after embedding (Lazzerini et al. 2010; Roy and Laha 2015). Some studies have also shown that PSNR poorly correlates with the subjective quality (Huynh-Thu and Ghanbari 2012; Tanchenko 2014); however, PSNR is still extensively used in evaluating the noise/denoising performance (Ong et al. 2006; Yoo and Ahn 2013), although PSNR as a quality metric that is constantly debated, in that it either is/is not relevant or is wrongly used as a quality metric. It is given by the following equation:

15 $$PSNR\left( {X,X^{\prime}} \right) = 10\log_{10} \frac{{X_{peak}^{2} }}{{\lambda_{j}^{2} }}$$

where $\lambda_{j}^{2}$ is defined as $\lambda_{j}^{2} = \left( {\frac{1}{Z}} \right)\sum\nolimits_{i = 1}^{Z} {\left( {y\left( i \right) - y^{\prime}\left( i \right)} \right)^{2} }$ where Z is the length of the host audio, and $y\left( i \right)$ is the magnitude of the audio *X* at time *i.* Similarly, $y^{\prime}\left( i \right)$ denotes the magnitude of the watermarked audio $X^{\prime}$ at time *i*. $X_{peak}^{2}$ denotes the squared peak value of the host audio. The higher PSNR means that the watermarked image is similar to the original image file (Chen et al. 2011).

Normalised cross-correlation (NCC) is the next metric that is used to evaluate the correlation between the extracted and the original watermark (Debella-Gilo and Kääb (2011); it is given by

16 $$NCC\left( {W,W^{\prime}} \right) = \frac{{\sum\nolimits_{i = 1}^{M} {\sum\nolimits_{j = 1}^{M} {W\left( {i,j} \right)W^{\prime}\left( {i,j} \right)} } }}{{\sqrt {\sum\nolimits_{i = 1}^{M} {\sum\nolimits_{j = 1}^{M} {W^{2} \left( {i,j} \right)} } } \sqrt {\sum\nolimits_{i = 1}^{M} {\sum\nolimits_{j = 1}^{M} {W^{{{\prime }2}} } \left( {i,j} \right)} } }}$$

In the above equation, W and *W*′ constitute the original and the extracted watermarks, whereas *i*, *j* represent the indices in the binary watermark image. If *NCC (W, W*′*)* is close to 1, then the correlation between *W* and *W*′ is very high. On the other hand, if it is close to zero, then there is very low correlation. Utilising the above equations, selected image processing/enhancement operations are performed to assess the robustness of the technique.

## Result and discussion

**Fig. 5:**
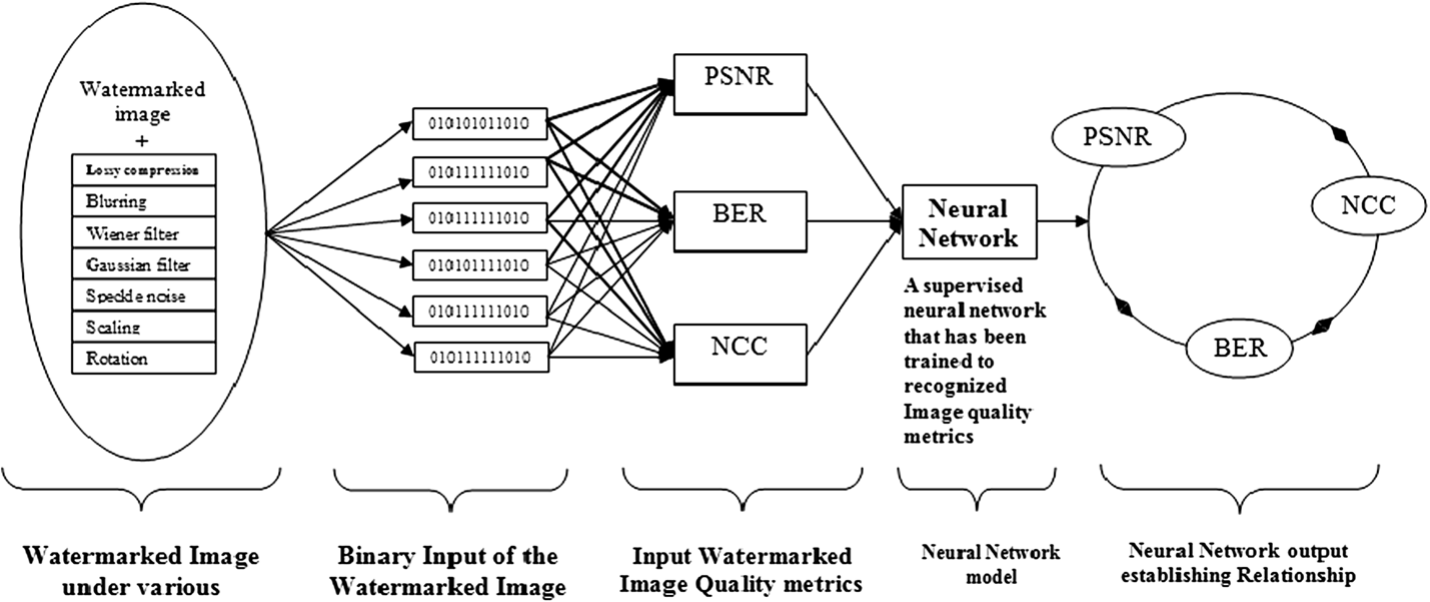
The proposed framework of the watermarked MLPNN modelling processes

Figure 5 shows an image (host 5) with the images after applying seven selected attacks without embedding any watermark. The comparison between all of these images is displayed in Table 1; these results were accomplished by calculating the average of the pixel changes as a percentage (*P*) according to Eq. 17.

17 $$\bar{p} = \frac{{\sum\nolimits_{i = 1}^{N} {\left| {P_{i}^{'} - P_{i} } \right|} }}{N} \times 100\;\%$$

where P is the original pixel, P′ is the pixel after applying the attack, and N is the total number of pixels.

**Table 1:** The effectiveness of the attacks on the image

Attacks	Average of pixels change % according to Eq. 17
Lossy compression	2.8896
Blurring	7.3548
Wiener filter	2.7365
Gaussian filter	1.5735
Speckle noise	5.9014
Scaling	5.6076
Rotation	5.7719

From the above results, it is clear that the effect of the Gaussian filter on the image (the average pixel change) was the least, and the effect of blurring was the highest.

The PSNR and NCC using the proposed method for the 1st to 8th bit-planes, and all of the possible bias values (X), were calculated after applying the chosen attacks. Although some attacks were found to improve the quality of the image (such as filtering and compression), others were found to destroy the image (such as blurring and noise). The results prove that the best watermarked image quality (the highest PSNR) was found when the bias value was the minimum (X = 0, the nearest pixel to the original), while the worst quality occurred when the bias value was the maximum. On the other hand, the best robustness (the largest NCC) could be obtained with the maximum bias value (in the middle of the ranges), but the worst was when the bias value was at a minimum. We noticed that the high quality of the digital watermarking images yielded the same result with the minimum bias value, while the robust digital watermarking method gave the same result as with the maximum bias value. The rotation and scaling were also tested for each embedding for the different bit-planes and different bias values. The NCC value for rotation was approximately 0.7995 for all of the embedding, and the NCC value for scaling was approximately 0.8092 for all of the embedding. These results are similar to those obtained from the LSB method. In other words, if the pixel was at the edge or middle of the range in any of the bit-planes, these two attacks would not affect the robustness.

**Fig. 6:**
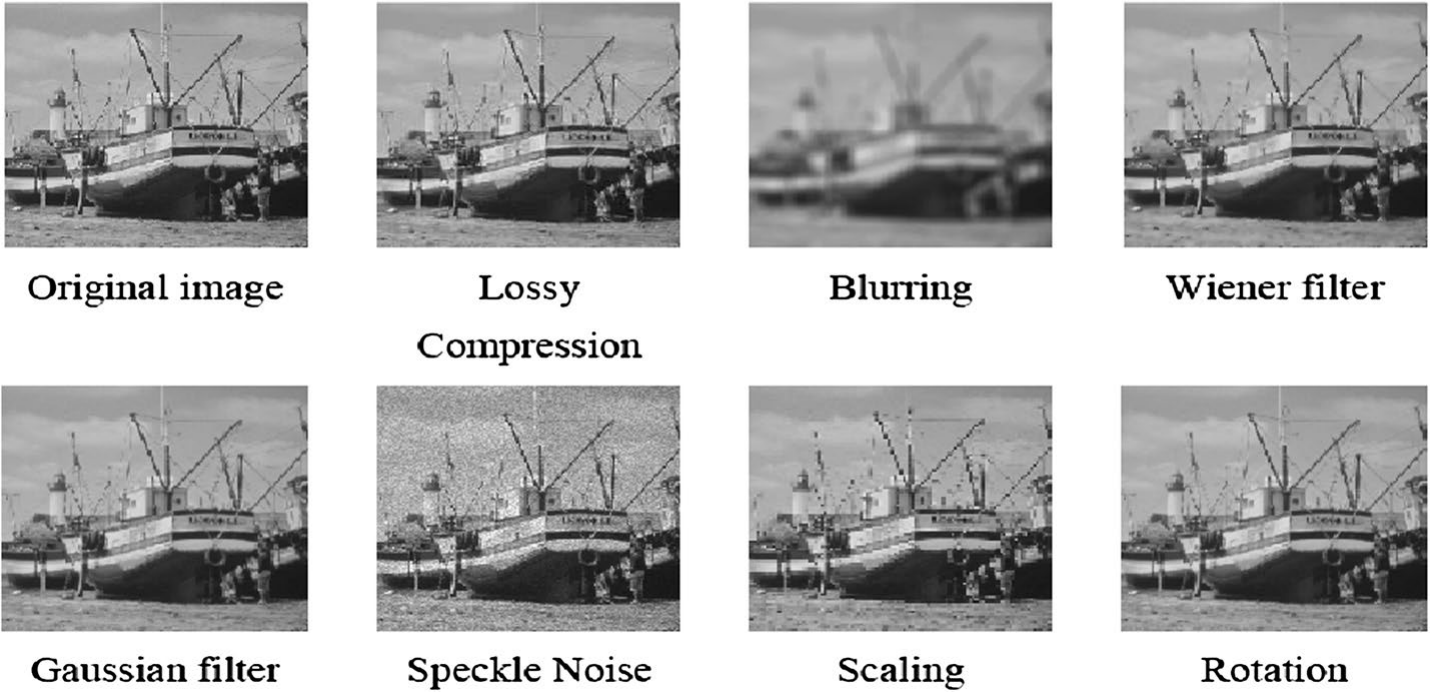
Images before and after applying different attacks

**Fig. 7:**
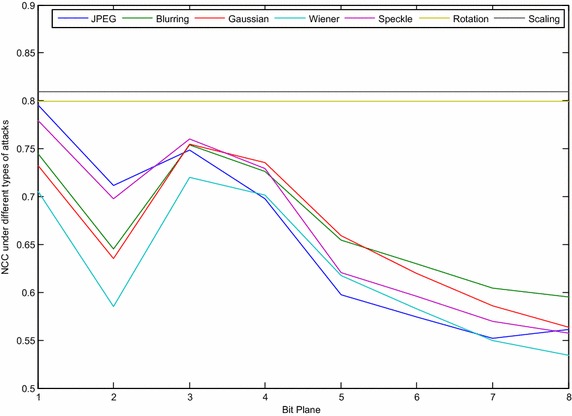
NCC value for the ISB method under different types of attack

The NCC values, after applying the chosen attacks for the ISB and LSB method for different bit-planes, are presented in Figs. 6 and 7 respectively.

**Fig. 8:**
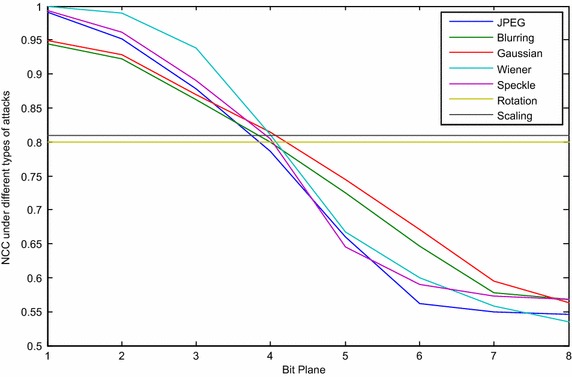
NCC value for the LSB method under different types of attack

From the above figures, it can be noted that the robustness in both methods has been improved by moving from the 8th bit-plane to the 1st, which occurs because the attack can change a small value (the last bit-planes) much more easily than a large value, such as in the first bit-planes. Although the watermarked image quality of the proposed method has been improved over the LSB method, the robustness was also found to have been reduced, as can be seen from the decrease in the NCC values. The difference between the NCC in the proposed method and the LSB method increases when moved from the 8th bit-plane to the 1st. Figure 8 can be considered as an introduction to reaching a compromise between the image quality and robustness. Similarly, Figs. 7 and 8 show that the results for rotation and scaling stay the same for different bit-planes, and they give the same NCC values as for the LSB in the proposed method. The reason is that both attacks are geometric transformation attacks, in which the intensities of the pixels are not affected by these attacks, while the locations of the pixels are changed.

**Fig. 9:**
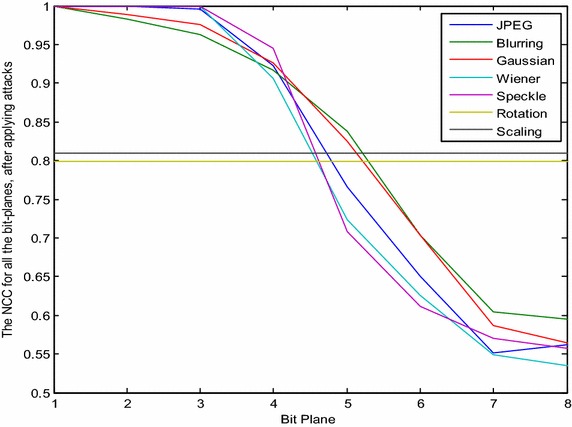
The NCC value of the ISB method for all of the bit-planes, after applying the chosen attacks

Further analysis was conducted by using every pixel and shifting them to the middle of the range in such a way that any modification on the pixel by attacks will minimally affect the selected bit. By choosing the watermarked pixel to be in the middle of the range, the same range is selected if the embedded bit is 1 and the selected bit of the original pixel value is also 1 or by embedding 0 if the selected bit of the original pixel is 0. In contrast, if the selected bit of the original binary pixel is not equal to the embedded pixel, then the middle of the previous or next range is selected. The new range is determined according to its distance from the original pixel value. In other words, if the nearest range to the original pixel is chosen, the quality of the watermark will be the best possible because the difference from the original pixel is the minimum. At the same time, if the middle of the range is chosen, the robustness will be the best possible that can be achieved. The reason is that the point is the farthest from the edge; thus, any attack will have a minimum effect on it. Note that there are two middle values because the number of pixels in the range is even; thus, the nearest value to the original pixel is selected to be the watermarked pixel. Figure 9 shows the extracted image (watermark 1) from host 1 using the proposed technique after applying the chosen attacks. All of the bit-planes were tested, starting from the 1st bit-plane (MSB) through the 8th bit-plane (LSB). The NCC was also calculated for each embedding. Note the similarity of the test for the LSB method presented in Fig. 8.

The NCC values of the extracted watermarks for the ISB method in all of the bit-planes, after applying the chosen attacks, are better than for the LSB method, evidently from the above result. The ISB method (which chooses the middle of the range to be the location of the watermarked pixel), especially in the first bit-planes that have large values in the range, prove to be an improvement on the LSB technique. This improvement is gradually decreased from the 1st to the 8th bit-planes. Moreover, it can be observed that for the 7th and 8th bit-planes (the least significant bits), the quality of the extracted logo is neither improved nor affected when using this method because of the small length of the range (the lengths of the 7th and 8th bit-plane are 2 and 1 respectively); therefore, there is no middle value, and the watermarked pixel is on the edge of the range. It can also be observed that the robustness does not improve for the rotation and scaling attacks because they are both considered to be geometric transformation attacks, which do not affect the intensity of the pixels. In contrast, they change the locations of the pixels. In other words, this method is more robust against image processing operations (compression, filtering, blurring and noise) that modify the intensity of the pixels.

As described in the previous sections, the location of the watermarked pixel was tested according to the range of each bit-plane. Thus, if the pixel value was located at the edge of the ranges, any small change by the attacks was found to move the pixel from one range to another, and the watermark could not be extracted. On the other hand, if the watermarked pixel was in the middle of the range, any effect on the pixel by attacks would then make it difficult to move the selected bit to another range, and the bit could be extracted correctly. The balance between robustness and the quality of the watermarked image was accomplished by positioning the watermarked pixel away from the edge of the range. The best bias value (the threshold value) between the middle and the edge of the range was found to survive against different types of attack and, at the same time, to retain the best image quality. Assume that the bias value (X) is the minimum distance from the position of the watermarked pixel P*′* to the edge of the range, which is closer to the original pixel. This arrangement means that if the distance from the pixel to the edge of the range is greater than the bias value, the position of the pixel will not change. In contrast, if the distance from the pixel to the edge of the range is smaller than the bias value, the position of the pixel will change to be as far as the bias value. The PSNR and the NCC for the proposed method of all of the possible bias values (X) were calculated after applying the chosen attacks, for all of the eight bit-planes. These calculated values were used to build the first MLPNN model for predicting the limiting values for PSNR and NCC.

**Fig. 10:**
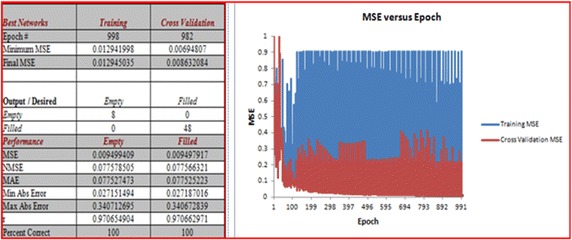
Result on zero bit results of embedding for all of the pixel value cases from 0 to 255

The two important metrics are the PSNR and NCC, which are taken as input vectors in Fig. 10. Hybridisation of unsupervised and supervised learning techniques was proposed and implemented using NeuroSolution 6.0 and Matlab 13b. The ISB technique that this research used takes the threshold value for an acceptable image quality in the 4th bit-plane (when the bias value is 6). It was observed that the difference between the watermarked pixel and the original pixel changed from 0 to 14 in all of the ranges of the bit-planes, except for the first and last, where the differences changed from 0 to 22. This finding means that if the original pixel value is 0 and the embedded bit is 1, the watermarked pixel will be 22 (the minimum of the next range + 6 = 16 + 6 = 22). The same result will be obtained if the original pixel value is 255 and the embedded bit is 0: the watermarked pixel will be 233 (the maximum of the previous range − 6 = 239 − 6 = 233); thus, the difference between the two pixels is 22. The result of a neural network model of the watermarked pixel value (P*′*) when embedding 0 into all of the option cases of the original pixel values (P) from 0 to 255 is shown in Fig. 10. The desired output obtained during the embedding also shows a good result in the NN model by giving MSE empty = 0.009, MSE filled = 0.009, NMSE empty = 0.07, NMSE filled = 0.07, MAE empty = 0.07, MAE filled = 0.07, Min Abs Error empty = 0.02, Min Abs Error filled = 0.02, Max Abs Error empty = 0.03, Max Abs Error filled = 0.03, r empty = 0.9, and r filled = 0.9. The overall performance of the output/desired value is 100 %.

**Fig. 11:**
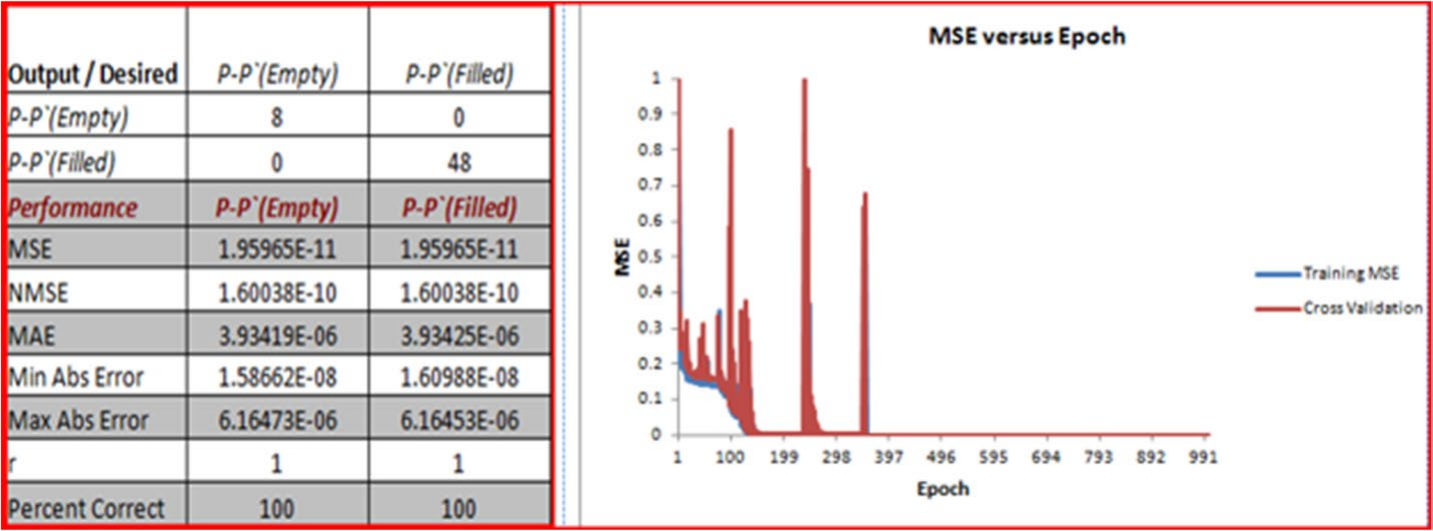
Result on one bit results of embedding to all pixel value cases from 0 to 255

The result of the NN model of the watermarked pixel value (P*′*) when embedding 1 into all of the cases of the original pixel value (P), from 0 to 255, is shown in Fig. 11. The desired output obtained from the embedding also shows a good result in the NN model by giving MSE empty = 1.9, MSE filled = 1.9, NMSE empty = 1.6, NMSE filled = 1.6, MAE empty = 3.9, MAE filled = 3.9, Min Abs Error empty = 1.5, Min Abs Error filled = 1.6, Max Abs, Error empty = 6.1, Max Abs Error filled = 6.1, r empty = 1, and r filled = 1. The overall performance of the output/desired value is 100 %.

**Fig. 12:**
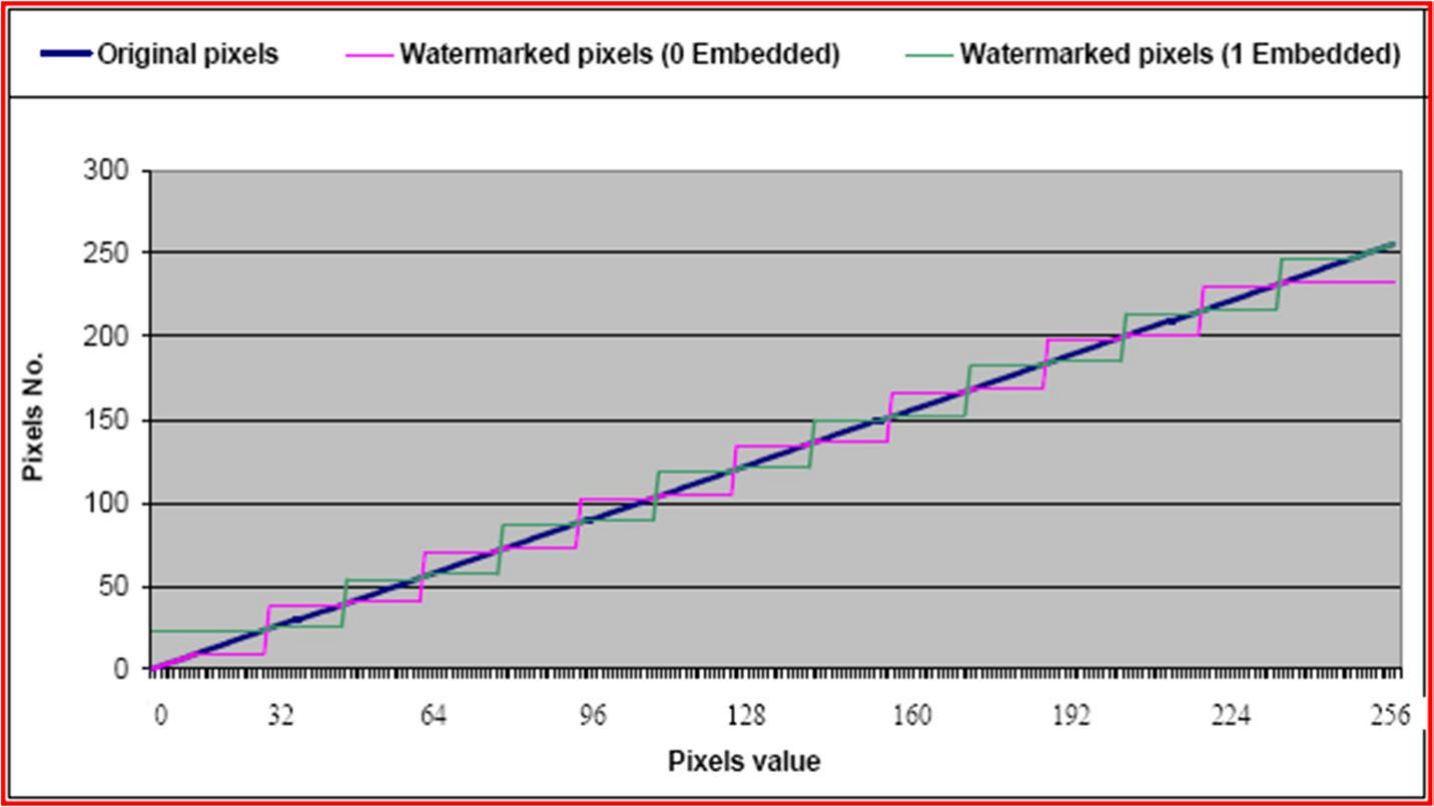
The watermarked pixel when embedding 0 and 1 to all possible pixel values from 0 to 255

The difference between the original pixels (P) and the watermarked pixels (P*′*) presents the error of the watermarked pixels, which is the quality of the distortion value of the watermarked image. Figure 12 presents the watermarked pixel of all cases of the original pixel values from 0 to 255, when 0 or 1 is embedded.

**Fig. 13:**
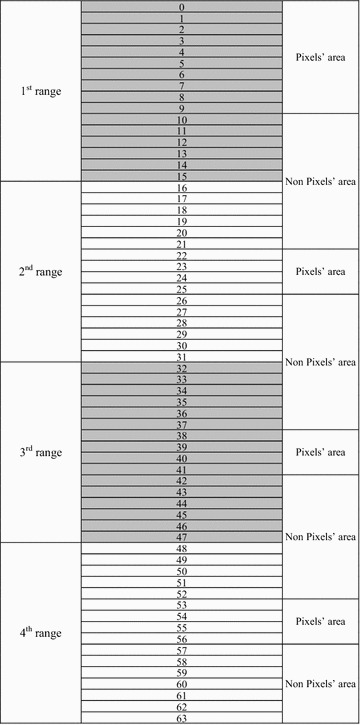
The new distribution of the image for the watermarked images by the proposed method

Figure 12 shows that the watermarked pixel value is moved around the original pixel value, and in some cases, they are identical [where the distance between the original pixel value and the edge of the range (the bias value) is 6 or above]. This arrangement means that if the embedded bit is equal to the original, and the pixel located at the edge of the range or having a distance of less than 6 (from the edge of the range), the watermarked pixel will move to be at a distance of 6 from the edge, and the difference between the watermarked pixel and the original pixel will range from 1 to 6. In contrast, if the original pixel is located as far as 6 or is greater than 6, the watermarked pixel will remain the same as the original. If the embedded bit is not equal to the original bit, the watermarked pixel will be located in the next range, and the difference between the watermarked pixel and the original should change between 14 and 7, while the difference is supposed to be between 7 and 22 if the original pixel is located at the first or the last of the range. These remarks are further illustrated in Fig. 13.

**Fig. 14:**
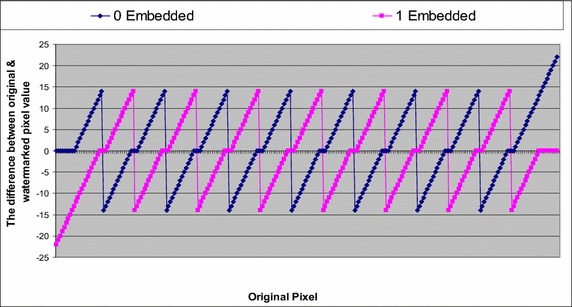
The difference in the values of the original and watermarked pixels, when embedding 0 and 1 to all of the possible pixel values from 0 to 255 for the bias value of 6

The difference between the value of the original pixel and the watermarked pixel (the error) is illustrated in Fig. 14 when 0 and 1 are embedded in all cases, from 0 to 255.

**Fig. 15:**
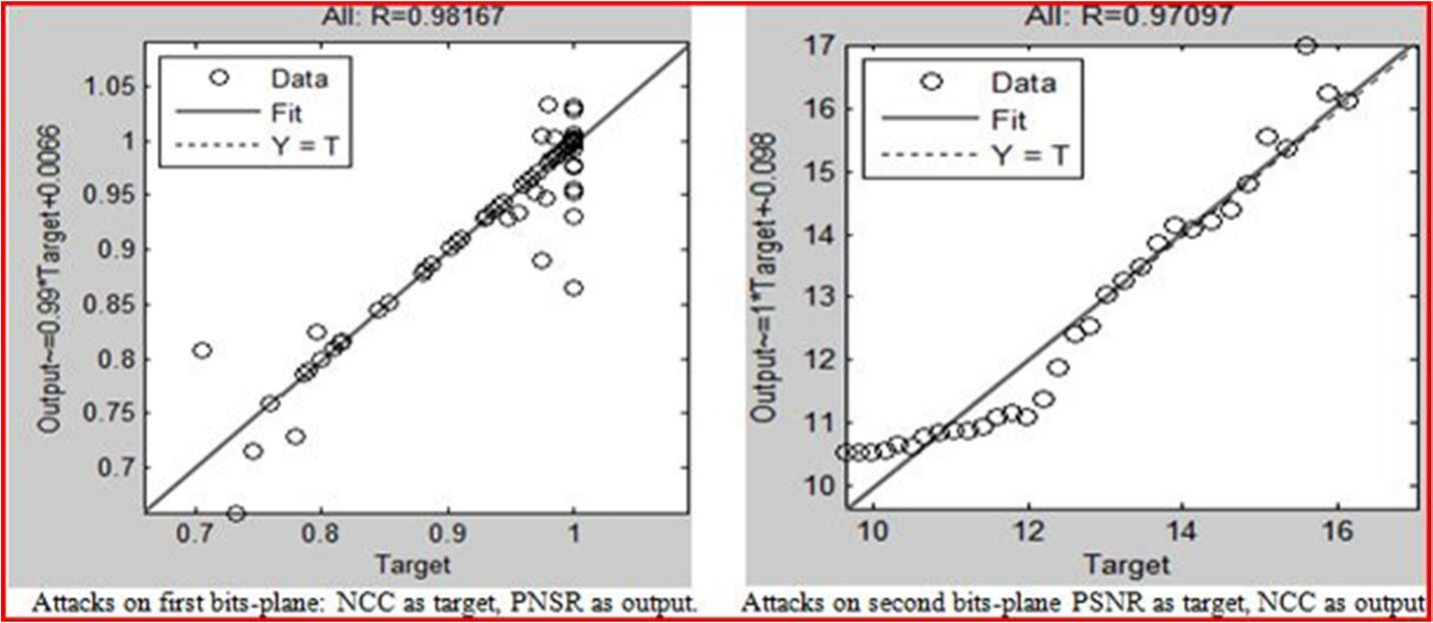
Result of the attacks on the first bit-planes

**Fig. 16:**
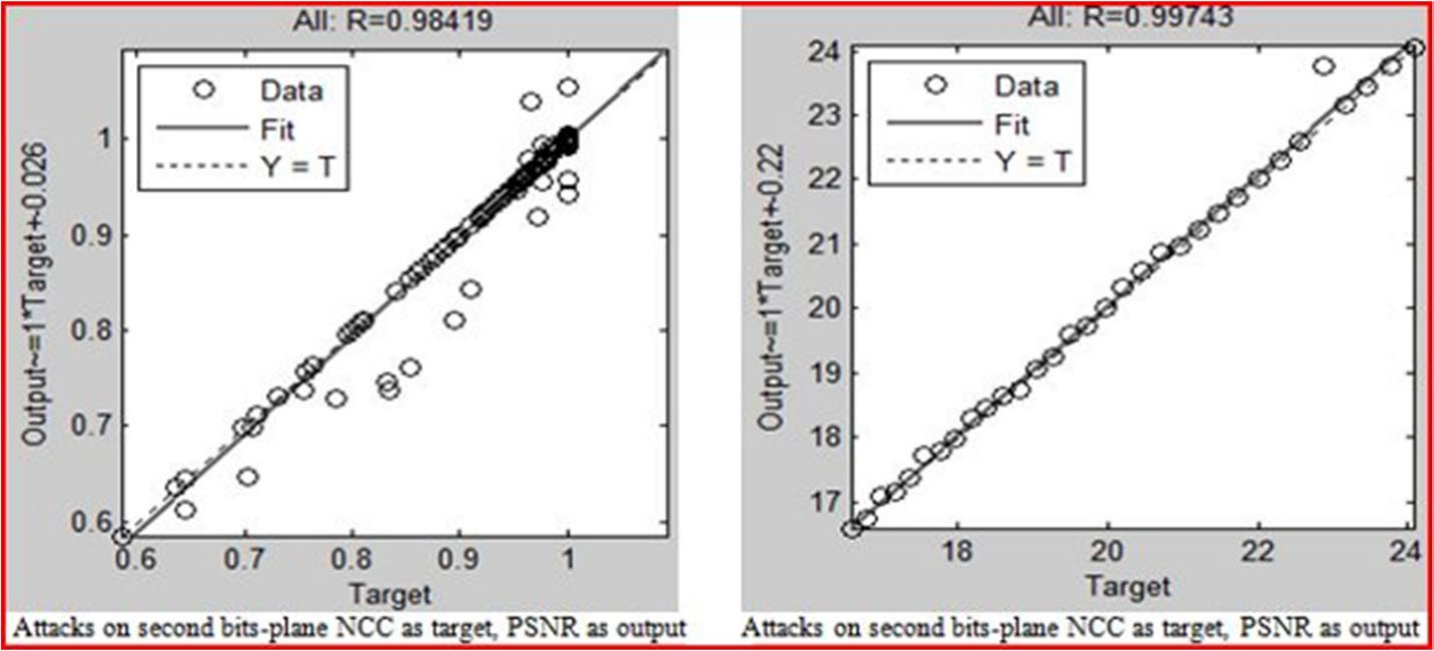
Result of the attacks on the second bit-planes

**Fig. 17:**
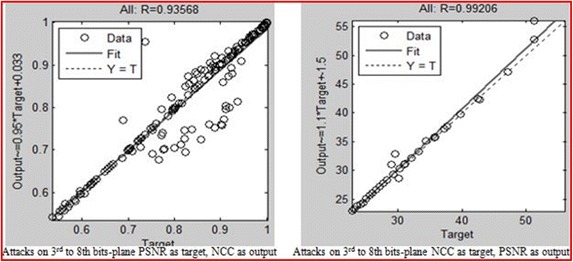
Result of the attacks on the third to the eighth bit-planes

The NCC and PSNR values for different attacks after embedding the watermarks within hosts using the proposed method were also modelled by the NN. The results of the neural network model of a situation in which the desired output is PSNR and the output is NCC and vice versa for embedding within the 1st to 8th bit-planes are presented in Figs. 15, 16 and 17. The aim is to validate the results obtained after applying the attacks, which shows that the NCC for all attacks was found to be very close to 1, and the PSNR all greater than 30 dB. This arrangement reflects a robust watermarking technique with high quality. The NN models for the embedding in the entire bit-plane show that those entire embedding models are fit with r values that are greater than 0.93 for all cases, after considering that MSE is the average squared difference between the outputs and targets. Lower values are better, and zero means no error.

## Discussion

This study has revealed the best pixel value (the threshold value) between the middle and the edge of the ranges, which survives against different types of attack, and at the same time maintains minimum image distortion. By testing all of the possible positions of the pixel between the edge and the middle of the range, and by considering an acceptable image quality of greater than 30 dB with the PSNR, the best normalised cross-correlation was found to be in the 4th bit-plane, when the bias value was 6. The bias value is the distance from the position of the watermarked pixel to the edge of the range. To validate the metrics for robustness, an MLP neural network model was built and trained for the entire embedding of the watermark and the attacks on watermarked images, with the desired output. The model fitness to this parameter indicates a very good result, by which we confirmed that every PSNR and NCC obtained during this experiment is fit. Although it was shown that the PSNR and NCC values fluctuate at certain times, and that the picture sometimes shows fluctuations at the same time, this arrangement means that there is no guarantee that when NCC indicates high values toward 1, the PNSR values will give a poor result. The fact remains that one goes up on one occasion higher and the other follows, and vice versa. Therefore, the ISB technique can be regarded as successful.

This study has shown that from the values of both PSNR and NCC, improving the robustness by partitioning the host image into blocks in the different sizes of 3, 5, 7 or 9 pixels is a breakthrough. For this purpose, the proposed method was applied (4th bit-plane at the bias value = 6), and the embedding was repeated for each bit of the watermark within all of the pixels in the block. The idea for repeating the embedding was to increase the robustness of the watermark system against all types of attack, specifically against the geometric transformation attacks. It was obvious that the capacity of embedding decreased by increasing the number of repetitions of the embedded bits. The results showed that the value of NCC increased with the increase in the embedding number for all of the attacks. The results yielded for the NCC became very close to 1 after nine repetitions, especially for rotation and scaling.

Similar to the above experiment, the image was partitioned into blocks of different sizes of 3, 5, 7 and 9 pixels. For every block, the average of the pixels was found, and the proposed method was applied to this average. The results showed that the NCC value improved with the increase in the size of the block for all of the attacks, including the geometric transformation attacks (rotation and scaling), which did not improve when the method was applied based on one pixel only. The idea behind the application of the proposed method, using blocks, was based on the assumption that the blocks were more resistant to attack than the single pixel, which was shown to be true. The result suggested that the block of three pixels was almost sufficient for all of the attacks, except for rotation and scaling, where a larger size of block might be needed.

Comparing the two methods, it was found that the one that made use of the average of the pixel values was better than that based on repetition, for all of the attacks, except for the rotation and scaling, which was indicated to improve extensively by means of the repetition method. For both methods, the results of the PSNR showed that the quality of the images was suitable for applying the proposed method based on any size of block. This arrangement occurs because the embedding within the 4th bit-plane, at the bias value of 6, is the threshold value that maintains the quality of the image. In other words, applying the proposed method based on individual pixels or blocks of pixels changes the pixels but has some restrictions and adjustments, which are important for the quality.

Furthermore, good outcomes were found to be maintained for robustness and capacity by embedding the most important information within large blocks and the other information within small-sized blocks because the most significant bits are more important for the quality of the image than are the least significant bits. A few examples were given using both methods, namely, repeating embedding in a block and embedding based on the average of the pixel values. The results showed that with this method the NCC was generally better than embedding within one size of the blocks with the same capacity.

For more data embedding, a new method based on PVD was applied. The image was partitioned into blocks, each containing 3 × 3 pixels. Three critical pixels were found (the maximum pixel value, the minimum pixel value and the third value chosen from the remaining seven pixels) in such a way that it was the farthest point from these two pixels. For the three points, only one bit was embedded within each 4th bit-plane at the bias value of 6. A high embedding capacity was embedded within the other six points. For this purpose, a range table was designed, and the embedding process was applied to all of the blocks, from right to left and from top to bottom. An imaginary plane was also passed through the above three critical pixels. The idea of using this plane was to embed more data within the edge areas because the HVS is less sensitive to distortions around these areas compared to the smooth areas, as well as acting as a smoothing element. Using the imaginary plane, the watermarked image would be very close to the original image. The characteristics of this plan should not be changed after embedding the message, and the same characteristics could be used to evaluate the embedded capacity in the extracted module. The total capacity obtained was in the range 22–26 %. This is considered to be a very high embedding capacity.

## Conclusions

The ISB techniques proposed have been proven to be robust based on two facts: (1) the experimental results of embedding and application of seven different attacks; (2) the model fitness of the MLP neural network that was trained for the robustness metric and that tested the results of the first experiment, yielding a notably good outcome. These findings show that PSNR and NCC used for measuring the robustness of these techniques perform well, and their behaviours were revealed to correspond to one another. The evaluation of the attacks that this paper presents is concerned with the spatial digital watermarking method based on the bit-plane model. This technique shows an improvement in the robustness of the digital watermarking system. Every pixel used for embedding is moved to be in the middle of the range (i.e. far away from the edge of the bit-plane), in such a way that any change in the pixel by the attacks will minimally affect the selected bit. Using the proposed method, which selects the middle of the range as the location of the watermarked pixel, the NCC of the extracted logo is better than the LSB method, especially in the first bit-planes, which comprise a large range of the table. This improvement was found to decrease gradually from the 1st to the 8th bit-planes. It was also found that robustness does not improve for the rotation and scaling attacks because both are considered to be geometric transformation attacks, and these attacks do not affect the intensity of the pixels because they change the locations of the pixels. In other words, the proposed method is more robust against image processing operations such as compression, filtering, blurring and noise, which modify the intensity of the pixels rather than their locations.
